# Role of Perivascular Adipose Tissue in Vein Remodeling

**DOI:** 10.1161/ATVBAHA.124.321692

**Published:** 2025-03-13

**Authors:** Nicky Kruit, Thijs J. Sluiter, Margreet R. de Vries

**Affiliations:** Department of Surgery (N.K., T.J.S., M.R.d.V.), Leiden University Medical Center, the Netherlands.; Einthoven Laboratory for Experimental Vascular and Regenerative Medicine (N.K., T.J.S., M.R.d.V.), Leiden University Medical Center, the Netherlands.; Department of Surgery, Brigham and Women’s Hospital and Harvard Medical School, Boston, MA (M.R.d.V.).

**Keywords:** adipokines, arteriovenous fistula, chemokines, cytokines, inflammation

## Abstract

Perivascular adipose tissue (PVAT) plays a crucial, yet underexplored, role in vein remodeling, which occurs after bypass surgery using a venous graft or creation of arteriovenous fistulae for hemodialysis access. PVAT exhibits significant heterogeneity in phenotype and tissue composition depending on the vascular bed, as well as its anatomic location within the vasculature. Through the excretion of adipokines, cytokines, and chemokines, PVAT can shape the vascular response to local and systemic perturbations. Moreover, the active exchange of cells reinforces the bidirectional cross talk between the vessel wall and PVAT. In this review, we describe the role of PVAT in relation to postinterventional vein remodeling, specifically focusing on the effect of surgery on the PVAT phenotype. Moreover, we discuss the pathophysiological mechanisms that ultimately affect clinical outcomes and highlight the therapeutic potential of PVAT to improve vein remodeling.

**Highlights**

- Obesity and nutritional status shape the perivascular adipose tissue microenvironment.
- The distinct phenotype of the venous wall is greatly influenced by perivascular adipose tissue and contributes to differential remodeling responses in surgically created arteriovenous reconstructions.
- Understanding the role of perivascular adipose tissue in postinterventional vein remodeling may uncover therapeutic strategies to enhance the long-term success of venous bypass grafts and arteriovenous fistulas.
- Future research using advanced imaging and multiomics technologies is essential to delineate the distinct role of venous perivascular adipose tissue and uncover the molecular and cellular mechanisms driving its influence on vein remodeling, potentially leading to new therapeutic strategies for improving vein graft and arteriovenous fistula outcomes.

Perivascular adipose tissue (PVAT) surrounds the majority of blood vessels and is located adjacent to the tunica adventitia.^1^ Although PVAT has long been overlooked in vascular biology, it is currently recognized as a unique, paracrine and autocrine organ that actively maintains vascular tone and affects vascular (patho)physiology through the excretion of adipokines and vasoactive molecules.^2^ Moreover, in addition to adipocytes, PVAT harbors a multitude of other cell types, such as endothelial cells and immune cells, which actively contribute to vascular health and disease. The obesity pandemic has augmented the interest in PVAT biology, which has increased our knowledge on its role in arterial diseases, such as atherosclerosis. In contrast, significantly less is known about the role of PVAT surrounding the venous vasculature. This review summarizes current knowledge on venous PVAT, especially in relation to postinterventional vein remodeling as observed after venous bypass surgery and creation of arteriovenous fistulae (AVF) for hemodialysis access.

**Please see www.ahajournals.org/atvb/atvb-focus for all articles published in this series.**

## PVAT Phenotype and Composition

Initially believed to serve as supporting connective tissue for blood vessels, PVAT is nowadays acknowledged as a distinct adipose depot that controls vascular homeostasis through the release of adipokines and cytokines.^3^ PVAT-derived adipokines such as adiponectin^4^ and angiotensin II,^2^ along with vasoactive molecules like NO and reactive oxygen species, help regulate vascular tone. Nutritional status is crucial for maintaining PVAT function because imbalances such as obesity and diabetes can promote (chronic) inflammation, impairing its ability to maintain vascular homeostasis. In contrast, a healthy diet and lean body composition support normal PVAT function. Moreover, metabolic reprogramming of PVAT can be driven upon disease, modifying its secretory profile and cross talk with mural cells, such as vascular smooth muscle cells (VSMCs) and pericytes.^5^ The extent and type of paracrine signaling is also dependent on the type of adipocytes residing in PVAT. There are 3 distinct subtypes of adipocytes, brown, white, and beige, which differ in morphology and function. Brown adipocytes are specialized in combusting nutritional energy into heat due to their high concentration of mitochondria and the expression of *Ucp1* (uncoupling protein 1), while white adipocytes, characterized by marker genes such as *Leptin* and *Fabp4*, are typically of larger size and store nutritional energy in the form of triglycerides. Beige adipocytes are resident within white adipose tissue (WAT) depots and can acquire a brown adipose tissue (BAT)–like phenotype. This process is called browning and is initiated upon cold exposure, hormonal signaling, or β3-adrenergic signaling.^6^

While other adipose tissue depots have a homogenic phenotype, that is, either BAT or WAT, PVAT emerges to be highly heterogenic and is actually a mixture of white and brown adipocytes. Depending on the vascular bed, the ratio between white and brown adipocytes in PVAT can differ greatly.^7–10^ Venous PVAT predominantly contains white adipocytes, whereas arterial PVAT holds a higher proportion of brown adipocytes. Indeed, PVAT surrounding the saphenous vein consists of larger adipocytes compared with arterial PVAT of the internal thoracic artery, coronary artery, and aorta.^10^ In contrast to BAT, WAT is metabolically less favorable and more prone to inflammation.^11^ In mice, caval vein PVAT exhibits reduced expression of genes related to thermogenesis, AMPK (AMP-activated protein kinase) signaling and PPAR (peroxisome proliferator–activated receptor) signaling compared with PVAT of the thoracic aorta.^12^ These pathways are all implicated in BAT, thus underscoring venous PVAT has a WAT-like phenotype. Nevertheless, the distinction in venous and arterial PVAT as being either more WAT or BAT like has been demonstrated to be too simplistic.

The anatomic location, in addition to the type of vascular bed, also determines PVAT development, function, and morphology.^13–15^ Around the thoracic aorta, PVAT is predominantly composed of brown adipocytes, while white adipocytes are primarily found in abdominal aortic PVAT (aaPVAT). In mice, the decreased expression of BAT genes in aaPVAT coincides with increased expression of proinflammatory genes, including *Il1*β** and *Tnf*α**, compared with thoracic aortic PVAT (taPVAT), highlighting the relation between adipose phenotype and tissue composition.^16^ Whether the PVAT phenotype around various veins is significantly different remains to be investigated.

In addition to adipocytes, a large variety of other cells reside in PVAT,^5,12,17^ including B cells, T cells, macrophages,^18^ as well as (mesenchymal) stem cells (perivascular adipose-derived stem cells).^19^ These cells actively contribute to its regenerative and adaptive function, with perivascular adipose-derived stem cells being particularly important due to their ability to differentiate into smooth muscle cells.^19^ PVAT further harbors nerves, as well as blood and lymphatic vessels, which are involved in vascular regulation, nutrient transport, and immune responses. Cells of these vessels, including fibroblasts, endothelial cells, and smooth muscle cells, help regulate vascular function.^5^ Recent investigations into the cellular composition of human peripheral veins highlighted a complex network of endothelial cells forming the vasa vasorum microvessels, which facilitates monocyte recruitment and infiltration into the vessel wall.^20^ These findings underscore the dynamic interaction between PVAT and the vein wall. Notably, the inflammatory characteristics of veins and arteries differ considerably, shaping their distinct roles in vascular health. Multiomics revealed significant differences in cellular composition and remodeling programs of human peripheral veins compared with arteries.^21^ Veins exhibited a higher presence of immune cells, such as macrophages, driven by proinflammatory endothelial signaling, enhanced oxidative stress responses from fibroblasts, and expanded vasa vasorum network. This results in an increased inflammatory microenvironment within venous tissue, likely contributing to greater immune cell infiltration in venous PVAT compared with its arterial counterpart. Additionally, extensive extracellular matrix remodeling and collagen production by venous fibroblasts further amplify immune cell recruitment and retention, underscoring the unique inflammatory and reparative processes within venous PVAT. Congruent with the distinct adipocyte types, the abundance and phenotype of these cells contribute to the overall structure and function of PVAT, which varies across different vascular beds.

## PVAT Plasticity

PVAT exhibits great plasticity and rapidly responds to changes in the circulation and blood vessel microenvironment. Dietary habits, particularly the consumption of Western-type diets, which are rich in fat and sugar, have a substantial impact on overall vascular health, including PVAT. Interestingly, murine aaPVAT and taPVAT were differentially affected by diets that were either high in sucrose or fat, indicating that dietary alterations yield disparate responses between distinct PVAT beds.^22^ High-sucrose feeding primarily affected aaPVAT by increasing adipocyte size and promoting infiltration of proinflammatory macrophages, whereas high-fat diet (HFD) induced similar changes in taPVAT. Moreover, both diets reduced UCP1 expression (indicating whitening of adipose tissue) in taPVAT, but not in aaPVAT, highlighting the heterogeneity of PVAT across vascular beds. Mechanistically, Notch signaling mediates whitening of taPVAT, which was characterized by suppression of thermogenic genes, as well as increased lipid deposition, transcription of *Leptin*, and inflammation.^23^ In contrast, 5-week 30% caloric restriction suppressed Notch signaling and reduced lipid droplet size, emphasizing the plasticity of perivascular adipocytes in response to nutritional changes.

PVAT, however, does not solely respond to dietary alterations. In mice, endovascular injury triggers infiltration of macrophages into both the vessel wall and its PVAT.^24^ Interestingly, rapidly after injury, increased expression of UCP1 was found in PVAT, indicative of browning, which was facilitated by the infiltrating macrophages. Moreover, the acquired BAT-like phenotype aims to limit the adverse effects of injury by promoting a more reparative PVAT phenotype. Indeed, the extent of damage to the arterial vessel wall was increased when macrophages were depleted or essential BAT genes (including *Prdm16*) were absent, indicating bidirectional cross talk between blood vessels and PVAT. This interplay is orchestrated by the vasa vasorum that enables exchange of cells, adipokines, and other excreted factors.^25,26^ Interestingly, browning of taPVAT is also observed after surgical injury to inguinal adipose tissue, demonstrating that surgery induces local and distant browning.^27^ Moreover, it establishes a significant role for PVAT in shaping responses to local and systemic perturbations and underscores its role in maintaining vascular homeostasis.

## Venous Conditions and Remodeling

While the perivascular microenvironment has been well studied for arteries, demonstrating an active role of PVAT in atherogenesis, the role of venous PVAT remains underexplored. While (perivascular) adipose tissue actively contributes to classic venous disease such as varicose veins^28^ and deep venous thrombosis,^29^ this review focuses on the role of PVAT in postinterventional vein remodeling, as observed after bypass surgery using venous grafts or surgical creation of a vein-to-artery anastomosis (AVF) for hemodialysis access.^30^ After these surgical arteriovenous reconstructions, the structural and cellular adaptations of the venous wall to perturbed flow that determine successful or adverse vein remodeling are largely shaped by PVAT.

Despite their ubiquitous use to revascularize hypoxic tissue downstream of atherosclerotic lesions, a large number of vein grafts fail as a result of adverse remodeling and accelerated buildup of advanced atherosclerotic lesions.^31^ Similarly, creation of an AVF is the preferred mode of hemodialysis access in patients with end-stage kidney disease, in spite of a failure rate over 50% within 1 year after surgery.^13^

After bypass surgery, the increased arterial pressure together with the disturbed blood flow cause maximal distension of the vein, resulting in cellular damage due to mechanical stretch. In AVFs, the altered flow hemodynamics in the outflow vein predisposes intimal hyperplasia around the anastomosis, while the flow over the AVF also predicts maturation.^32^ Specifically for AVFs, different configurations, such as artery to vein rather than vein to artery, aimed to optimize blood flow have been demonstrated to increase patency.^33^ The perturbed hemodynamics result in activation and partial denudation of the endothelium, exposing the subendothelial extracellular matrix, which instigates thrombosis.^31^ Furthermore, the surgical injury and loss of endothelial barrier function trigger infiltration of leukocytes into the vessel wall, which drives local inflammation and mesenchymal cell proliferation.^34^ This leads to thickening of the vessel wall, which is required to withstand the increased hemodynamic forces. Inadequate dilatation and thickening of the vein causes AVF nonmaturation, driving early failure.

Regularly, the inflammatory and proliferative responses are accompanied by a phenotypic switch of VSMCs from a contractile to synthetic phenotype.^35^ The migration of VSMCs to the intima and their subsequent proliferation leads to intimal hyperplasia. Additionally, endothelial cells also contribute to intimal hyperplasia through endothelial-to-mesenchymal transition in both vein grafts and AVFs.^36^ Interestingly, chronic kidney disease augments endothelial-to-mesenchymal transition and increases AVF failure.^37^ In vein grafts, the hypercholesterolemia that frequently occurs in these patients induces foam cell formation, leading to accelerated development of atherosclerotic plaques.^31^ Over time, when the intimal layer thickens at the expense of the lumen, this results in adverse vein remodeling, which drives venous bypass and late AVF failure.^38^

Animal models, particularly murine models, have provided valuable insights into the mechanisms underlying vein graft failure and PVAT involvement. However, it is essential to recognize the limitations of these models in directly translating findings to human condition. Key differences include variation in wall structure, cellular composition, and remodeling responses, which are affected by VSMC migration and proliferation, pronounced adventitial migration of fibroblast in mice, and extracellular matrix remodeling.^31^ Additionally, differences in hemodynamic changes lead to distinct vascular remodeling patterns. Despite these limitations, both clinical and preclinical evidence implicates PVAT as a critical regulator of postinterventional vein remodeling.^12,39–42^

## PVAT in Vein Remodeling

### No-Touch Technique

In venous bypass surgery, PVAT used to be routinely stripped together with the adventitial layer during surgical harvesting of the vein. Clinical observations of conduit spasm during removal of the surrounding tissue have led to the development of the so-called no-touch technique. This approach leaves the vein and the perivascular tissue intact to minimize surgical injury and thus limits cellular damage.^43^ Endothelial integrity is almost totally preserved when veins are harvested with the no-touch technique, while conventional stripping induces significant damage to the luminal endothelium.^43,44^ Additionally, the production of eNOS (endothelial NO synthase) by the luminal endothelium is maintained by the no-touch technique.^44,45^ Interestingly, PVAT expresses eNOS and serves as a major donor of NO in saphenous vein grafts, supporting vasorelaxation and reducing spasm. As a result, PVAT plays a crucial role in vasocontraction and vasorelaxation of vein grafts, even in the absence of luminal endothelium.^44,46^ This, therefore, also explains the preserved vasocontractile and vasorelaxant function of venous conduits when harvested by the no-touch technique.^47^

Clinically, the no-touch technique improves vein graft patency rates. Meta-analyses trials indicated that the no-touch technique reduces the number of vein graft occlusions compared with conventional harvesting at both 1 year,^39^ 8 years,^48^ and 16 years^40^ post-surgery. A randomized clinical trial recapitulated these results, demonstrating reduced angina pectoris recurrence and graft occlusions in the no-touch group.^41^ Others highlighted the impact of no-touch technique in preventing postoperative saphenous vein graft expansion, minimizing diameter mismatch between the graft and coronary circulation. This accentuates the importance of PVAT in vasorelaxation and vasocontraction.^49^ Taken together, these data underscore the importance of quality of the vein and PVAT at baseline for venous bypass surgery outcomes.

### Body Mass Index

Vein and PVAT quality are greatly affected by nutritional status. In an Asian population, the protective effect of no-touch harvesting was lost in patients with body mass index (BMI) >27 kg/m^2^.^41^ A similar effect was observed in a European cohort, in which no-touch harvesting appeared protective in patients with BMI <30 kg/m^2^, while this apparent effect was lost in patients with BMI >30 kg/m^2^, indicating a phenotypic switch in overweight and obese patients, although the low sample size limited statistical power.^39^ Correspondingly, a recent systematic review suggests an association between obesity and poorer AVF maturation outcomes, reduced primary patency, as well as higher reintervention rates.^50^ Mechanistically, excess adipose tissue surrounding the vein and (brachial) artery^51^ has also been postulated to limit vessel wall dilatation, which is required for AVF maturation. Altogether, these results indicate that PVAT may exert protective or adverse effects on vein remodeling depending on its phenotype.

### Inflammation

The surgical arteriovenous reconstruction induces, among others, an inflammatory response. While surgery influences PVAT signature and function, preoperative diet and nutritional status significantly govern the response to surgical injury (Figure). In mice, high-fat feeding before surgical trauma yielded increased expression of proinflammatory and matrix-degrading enzymes, which fuel inflammation since degradation products can act as damage-associated molecular patterns, compared with normal chow diet.^52^ Moreover, the inflammatory response to surgery was increased after HFD, while returning HFD-fed mice to normal chow diet 3 weeks before surgery reduced baseline levels of inflammation and prevented the aggravated response to injury. Furthermore, both before and after vein graft surgery, HFD accelerated adverse remodeling compared with mice fed a regular chow diet. This was accompanied by increased expression of CD11c (integrin αX) around the adventitia, suggesting outside-in infiltration of inflammatory cells via PVAT. The adverse effects of high-fat feeding were blunted in CD11c^−/−^ mice,^53^ suggesting inflammation dependence and underscoring PVAT plasticity, as well as its therapeutic potential.

**Figure.**
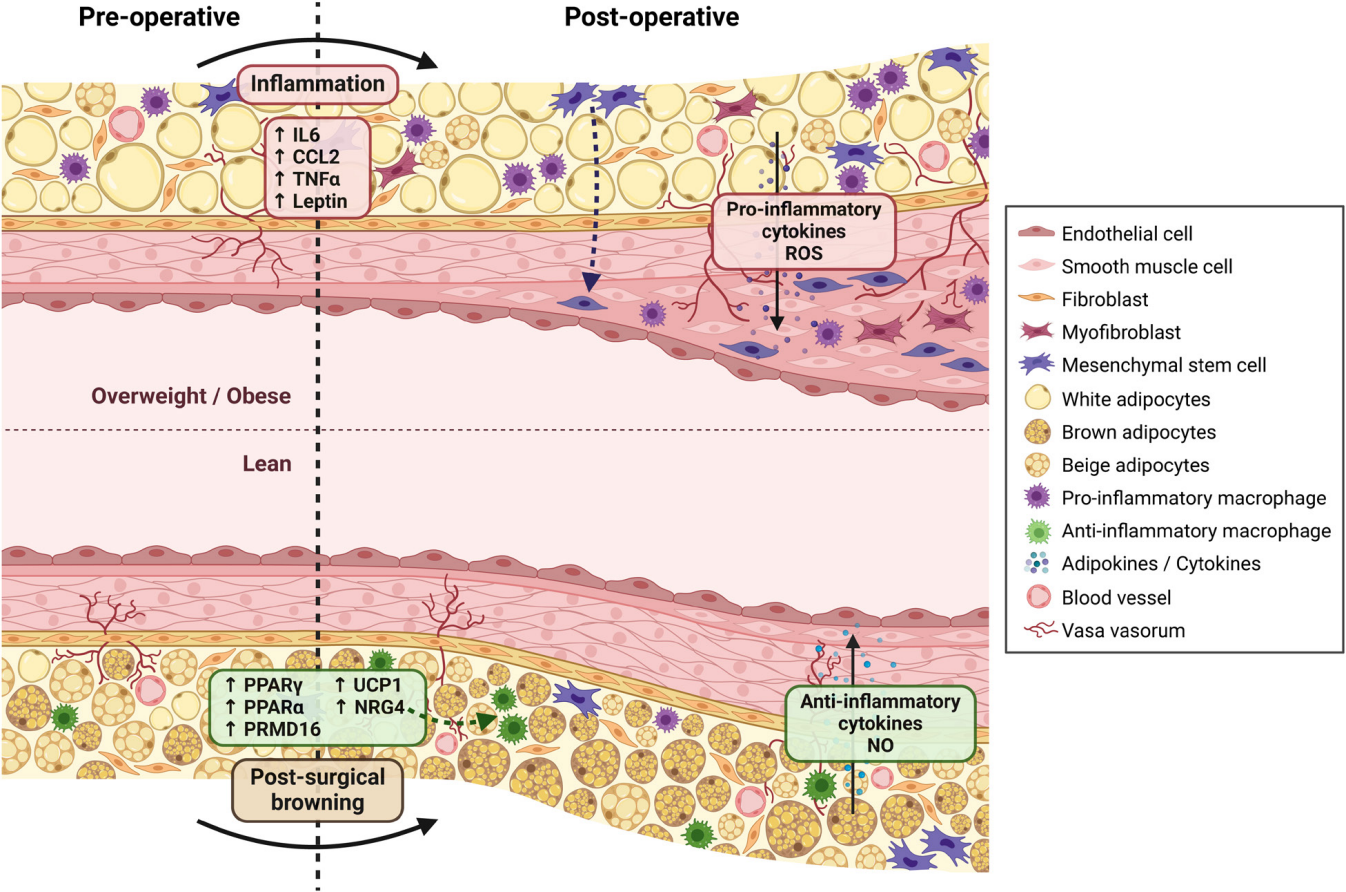
**Preoperative and postoperative changes in perivascular adipose tissue (PVAT) in lean versus overweight/obese conditions.** In lean individuals, PVAT acquires a brown adipose tissue (BAT)–like phenotype to mitigate the proinflammatory response to surgical injury. To facilitate vascular repair, NRG4 (neuregulin-4) expression is increased in PVAT adipocytes, which promotes transitioning of macrophages toward an anti-inflammatory phenotype. This is accompanied by release of anti-inflammatory cytokines and NO. In obese individuals, PVAT exhibits an increase in white adipocytes, elevated proinflammatory cytokines (IL-6 [interleukin-6], CCL2 [CC-chemokine ligand 2], TNFα [tumor necrosis factor-α], and leptin), and a higher presence of proinflammatory macrophages before surgery. Postoperatively, this inflammatory environment hampers browning of PVAT, which leads to the production of proinflammatory cytokines along with reactive oxygen species (ROS). This induces adverse vascular remodeling, which is mediated by enhanced vascular smooth muscle cell and mesenchymal stem cell migration and proliferation. NRG4 indicates neuregulin-4; PPAR, peroxisome proliferator–activated receptor; PRDM16, PR domain containing 16; and UCP1, uncoupling protein 1. Created in BioRender.

The earlier described effects of BMI and its relation to outcomes of venous bypass surgery were also observed in mice. When animals were fed an HFD, stripping of PVAT diminished adverse vein graft remodeling compared with no-touch. In contrast, the benefits of 7-day preoperative restriction of the sulfur amino acids (methionine and cysteine), without reducing calorie content, were dependent on PVAT.^12^ This methionine restriction (MetR) diet induced browning of venous PVAT, while also further enhancing it in taPVAT. More specifically, MetR increased expression of *Ppar*α** in PVAT adipocytes, suggesting PPAR signaling to mediate MetR-induced browning. Moreover, MetR significantly reduced the expression of proinflammatory and matrix-degrading enzymes in PVAT macrophages after vein graft surgery, underscoring the notion that preoperative diet impacts the response of PVAT to surgical injury. Interestingly, the browning response of PVAT upon MetR was maintained until 28 days of surgery and coincided with decreased proinflammatory/anti-inflammatory macrophage ratios. This shift toward a browning phenotype alters the PVAT microenvironment, potentially promoting the transition of macrophages from a proinflammatory toward an anti-inflammatory phenotype. In humans, MetR can be delivered as a semisynthetic diet for up to 16 weeks,^54^ highlighting its clinical translatability. Whether MetR can also mitigate adverse vein graft remodeling in humans, however, remains to be investigated. Identifying the molecular pathways that are responsible for the beneficial PVAT microenvironment and reduced inflammation, might aid in developing less invasive strategies compared with strict dietary alterations. For example, the alternative activation of macrophages after vascular injury appears to be regulated by *Nrg4* expression in beige adipocytes within PVAT.^24^ Local application of *Nrg4* small interfering RNA increased intimal hyperplasia, thus describing a potential mechanism through which PVAT influences vascular remodeling.

In a rabbit vein graft model, stripping of PVAT induced thrombosis and adverse vascular remodeling by increasing oxidative stress, endothelial activation, and inflammation.^42^ When PVAT-stripped vein grafts were supported by an externally placed 3-dimensional–printed stent, this reduced oxidative stress and expression of adhesion molecules on the endothelium, overall improving graft patency and limiting vein graft disease. The stent’s latticed structure offered opening channels that facilitated cross talk between the vein graft and its surrounding. Coating of these stents with metformin amplified the protective effects, due to increased PVAT regeneration and adventitial regeneration through expansion of the vasa vasorum, underscoring the importance of PVAT in vein graft remodeling.

In human AVFs, preoperative characterization of PVAT juxtaposed to the site of surgery demonstrated that a preoperative inflammatory phenotype, defined by elevated CCL2 (CC-chemokine ligand 2) levels, inversely correlated with maturation at 4 to 6 weeks after surgery.^55^ In a follow-up study, it was found that the negative correlation of IL (interleukin)-6 and CCL2 at baseline in venous PVAT with AVF maturation was statistically significant between 1 day and 2 weeks postoperatively but not at later time intervals.^56^ Moreover, there was also a negative correlation between leptin and flow increase from 2 to 6 weeks after surgery. Altogether, this indicates local venous PVAT phenotype at baseline significantly affects early AVF maturation by mediating outward remodeling. In another cohort, serum leptin was associated with BMI, diabetes, inflammation (high-sensitivity C-reactive protein and IL-6), as well as AVF failure 3 months after surgery. Even after correction for baseline age, sex, BMI, and other risk factors, leptin was still strongly correlated with AVF failure, thus potentially serving as a surrogate marker of inflammation.^57^ Overall, these data demonstrate that PVAT phenotype, and specifically inflammation, at baseline is a critical determinant of outcomes after arteriovenous surgery.

### PVAT-Derived Mesenchymal Stem Cells

The infiltration of leukocytes into the vessel wall and PVAT, together with the excretion of matrix-degrading enzymes, catapult adverse postinterventional vein remodeling by allowing migration of VSMCs from the media to the intima, followed by partial dedifferentiation and excessive proliferation.^38^ Recently, however, it was demonstrated that also mesenchymal stem cells (MSCs) from PVAT contribute to intimal thickening. In murine aortic PVAT, 2 distinct subsets of PVAT-derived MSCs were identified.^19^ Transplantation of fluorescently labeled PVAT-derived MSCs onto the adventitia of the vein graft immediately after surgery aggravated intimal thickening. Moreover, fluorescent signals were predominantly detected in cells expressing ACTA2 (α-actin-2), including VSMCs and MSCs, within the intima, revealing active contribution to intimal thickening, likely through VSMC differentiation. Mechanistically, transforming growth factor-β signaling mediated the transition of these PVAT-derived MSCs into VSMCs (upregulation of *Cnn1*, *Tagln*, and *Acta2*), which concomitantly decreased the expression of BAT-related genes such as *Ppar*α**, *Pparγ*, *Prdm16*, *Cebpb*, and *Ucp1*.

Similarly, PVAT-derived stromal cells (PVASCs) were found to have reduced capacity of brown adipocyte differentiation during aging.^58^ This suggests aberrant secretion of BAT adipokines and cytokines upon vascular injury, indicating that aged PVASCs might lose their protective effect. Indeed, transplantation of PVASCs from old mice to the adventitia after vascular injury aggravated adverse (arterial) remodeling, whereas young PVASCs reduced this process.^58^ Interestingly, these PVASCs (which are also found in patients undergoing coronary artery bypass grafting) exhibited differentiation ability into multiple cell types and were found with a myofibroblast phenotype in the intima of injured arteries. When overexpressing PPARγ coactivator 1α, a protein that promotes brown adipocyte development, these cells attenuated perivascular collagen deposition, providing direct evidence for their involvement in postinterventional vascular remodeling. Moreover, it indicates that vascular injury–induced browning is not only mediated by existing adipocytes but also through differentiation of PVASCs, underscoring their potential as a therapeutic target. In murine AVFs, transplantation of human adipose-derived MSCs improved AVF remodeling at 7 and 21 days post-surgery by increasing lumen size and reducing neointimal hyperplasia, which was derived from a decrease in inflammation and proliferation. Interestingly, the MSCs were observed not only in the adventitial region, where they were transplanted, but also in the luminal endothelial layer.^59^ Similarly, transplantation of murine adipose-derived MSCs (harvested from epididymal fat) after percutaneous transluminal angioplasty in murine AVFs reduced inflammation and specifically induced phenotypic switching of macrophages toward an anti-inflammatory phenotype after percutaneous transluminal angioplasty.^60^ Moreover, the MSCs increased lumen area while limiting intimal hyperplasia leading to increased flow. Over time, the number of MSCs (that were GFP^+^ [green fluorescent protein]) decreased indicating that these cells are particularly important in the early postoperative period in mitigating the inflammatory response to surgical injury, thus facilitating AVF maturation.

These beneficial effects have led to initiation of a phase 1/2, single center, clinical trial (https://www.clinicaltrials.gov; unique identifier: NCT02808208), in which MSCs will be harvested from subcutaneous fat and transplanted onto 1-stage radiocephalic, or brachiocephalic fistulas.^61^ Preoperatively, patients will undergo extensive vein mapping, which will also allow for transplantation of a number of MSCs that is proportional to the diameter of the vein.

## Conclusions

A growing body of both clinical and preclinical research provides compelling evidence that PVAT plays a crucial role in postinterventional vein remodeling. PVAT is increasingly recognized as critical regulator of the vascular responses to surgery, shaping clinical outcomes in procedures such as bypass grafting and AVF creation. Depending on its phenotype, PVAT can exert protective or adverse effects through secretion of inflammatory and vasoactive factors, as well as through the exchange of stem cells and leukocytes.

Recent advances in imaging technologies, such as coronary computed tomography angiography and PVAT attenuation analysis,^62^ have enabled noninvasive assessment of the inflammatory state in (arterial) PVAT. While PVAT attenuation has been associated with vascular inflammation and plaque instability in coronary arteries,^63^ its application to venous PVAT remains largely unexplored. Future studies evaluating venous PVAT phenotype through similar approaches could provide critical insights into its role in vein graft outcomes, assisting in identification of patients who might benefit from targeted preconditioning strategies, such as dietary interventions, anti-inflammatory treatments, or (partial) stripping of PVAT during surgery. These approaches could help mitigate the adverse effects of inflamed PVAT on remodeling and improve graft patency.

Despite significant progress, several challenges remain in fully understanding the role of PVAT in vascular health and vein remodeling. The phenotypic diversity of PVAT, influenced by factors such as obesity and metabolic conditions, as well as variability in its function across different vascular beds, complicates its consistent characterization and clinical translatability. While current research has focused primarily on arterial PVAT, there is a lack of knowledge regarding the role of PVAT in postinterventional vein remodeling. Integration of advanced technologies, such as single-cell (spatial) multiomics, will provide a deeper understanding of the complex cellular composition, molecular pathways, and interaction with the vascular wall. These tools could enable the identification of novel therapeutic targets aimed at modulating PVAT to improve surgical outcome.

In conclusion, as our understanding of (venous) PVAT conditions evolves, it is clear that targeting PVAT-related pathway offers promising therapeutic potential for improving vein graft and AVF outcomes. Current and future research endeavors will further increase our understanding of PVAT, opening new therapeutic avenues to combat adverse vein remodeling and improve long-term patency.

## ARTICLE INFORMATION

### Acknowledgments

The Graphical Abstract and Figure were created using BioRender.com.

### Sources of Funding

This study received support from the Rembrandt Institute for Cardiovascular Science to M.R. de Vries and Personal LUMC Theme Grant to M.R. de Vries.

### Disclosures

None.
